# A Hydrolase-Rich Venom Beyond Neurotoxins: Integrative Functional Proteomic and Immunoreactivity Analyses Reveal Novel Peptides in the Amazonian Scorpion *Brotheas amazonicus*

**DOI:** 10.3390/ijms27031475

**Published:** 2026-02-02

**Authors:** Gisele Adriano Wiezel, Karla de Castro Figueiredo Bordon, Jonas Gama Martins, Viviane Imaculada do Carmo Custódio, Alessandra Kimie Matsuno, Rudi Emerson de Lima Procópio, Eliane Candiani Arantes

**Affiliations:** 1 Department of BioMolecular Sciences, School of Pharmaceutical Sciences of Ribeirão Preto, University of São Paulo, Ribeirão Preto 14040-903, SP, Brazil; gisele.wiezel@gmail.com; 2 Graduate Program in Genetics, Conservation and Evolutionary Biology (PPG GCBEv), National Institute for Amazon Research (INPA), Manaus 69067-375, AM, Brazil; jonasgama83@gmail.com; 3 Department of Pediatrics, Ribeirão Preto Medical School, University of São Paulo, Ribeirão Preto 14049-900, SP, Brazil; viviane.custodio@baraodemaua.br (V.I.d.C.C.); matsuno@fmrp.usp.br (A.K.M.); 4 Barao de Maua University Center, Ribeirão Preto 14090-180, SP, Brazil; 5 Graduate Program in Biotechnology and Natural Resources of Amazon, University of the State of Amazonas (UEA), Manaus 69065-001, AM, Brazil; rudiprocopio@gmail.com

**Keywords:** bioactive peptides, Amazonian scorpion, integrative venomics, cross-reactivity, phospholipase A_2_, hyaluronidase, drug discovery, venom evolution, biotechnological potential

## Abstract

The scorpion family Buthidae, renowned for its neurotoxin-rich venoms, dominates toxinology, while non-buthid venoms remain largely unexplored. Here, we present a comprehensive proteomic and biochemical characterization of the Amazonian chactid scorpion *Brotheas amazonicus* venom (BamazV), with emphasis on molecular complexity, proteolytic processing, and peptide diversity. Using an integrative venomics approach that combines molecular mass-based fractionation, reversed-phase chromatography, high-resolution mass spectrometry, N-terminal sequencing, and functional and immunological analyses, we reveal an unexpectedly complex venom profile enriched in high-molecular-weight components and extensively processed peptides, with more than 40 venom peptides sequenced by MS/MS and Edman degradation. The data provide evidence for non-canonical proteolytic events, including the generation of peptides from precursor regions not classically associated with mature venom components. In contrast to the venom of *Tityus serrulatus*, BamazV displays a “hydrolase-rich, neurotoxin-poor” profile, featuring a catalytically active Group III phospholipase A_2_ (BamazPLA_2_), a highly active hyaluronidase, metalloproteases, low-mass peptides, and potassium channel toxins. Our results suggest a hydrolytic prey-subjugation strategy, and limited cross-reactivity with commercial antivenom highlighted its distinct structural landscape. Overall, this study advances the understanding of venom evolution and proteolytic diversification in underexplored scorpion lineages, positioning *B. amazonicus* as a valuable model for investigating alternative venom strategies and identifying novel biotechnological scaffolds.

## 1. Introduction

Scorpion venoms play central roles in prey capture, digestion, and defense, being composed mainly of protein and small peptides. These features have contributed to the evolutionary success of scorpions for more than 400 million years [1,2]. Their venoms comprise a functionally diverse repertoire of molecules, including neurotoxins acting on sodium, potassium, and calcium channels involved in prey immobilization and neurophysiological modulation; antimicrobial and cytolytic peptides with membrane-disruptive properties; and enzymatic components such as phospholipases, proteases, and hyaluronidases that facilitate tissue diffusion and digestion [2,3,4,5]. In addition to their ecological functions, many of these components also represent valuable molecular structures for pharmacological and biotechnological applications, since venom-derived ion channel modulators, antimicrobial peptides, and enzyme inhibitors have historically contributed to drug discovery and the development of molecular tools.

The Brazilian Amazon presents an abundant scorpion fauna, containing 48 species of which 6 are considered medically important and belong to the genus *Tityus* [6]. This medical relevance is associated with the high incidence of human accidents recorded in the region and the severity of clinical manifestations, which can lead to fatal outcomes. Consequently, most studies on Brazilian scorpions have focused on *Tityus* spp. [7,8]. However, the venoms of Amazonian scorpions without medical importance may also represent rich and underexplored sources of bioactive molecules, although reports on these species are still particularly scarce [6,9].

*Brotheas amazonicus* (family Chactidae) is a dark-colored Brazilian Amazonian scorpion inhabiting *terra firme* forests (non-flooded zone), where it is commonly found under fallen trunks and in burrows in the ground, and whose biological and toxicological relevance has long been neglected [6]. The first study regarding its venoms was reported in 1994, addressing its LD_50_ and antigenic cross-reactivity with *Tityus* antivenoms [10]. To date, only 13 reports involving *B. amazonicus* or its venom have been published, including original articles, reviews, and conference abstracts [6,7,8,9,11,12,13,14,15,16,17,18,19]. Among these, six [11,12,14,18,19] investigated biological or enzymatic activities of *B. amazonicus* venom (BamazV) components, two [15,16] addressed cytogenetic aspects, and one focused on the species’ geographic distribution [13].

In this context, the present study aims to characterize the molecular composition and functional profile of BamazV and its major fractions through an integrated proteomic and functional framework. This approach combines ultrafiltration, reversed-phase chromatography, mass spectrometry, N-terminal sequencing, enzymatic activity assays, and antivenom immunoreactivity analyses to systematically assess the diversity, relative abundance, and biological properties of BamazV components. By linking venom composition to functional activities and immunological recognition, this study expands public databases on *B. amazonicus* venom peptides and provides a foundation for future pharmacological, biotechnological, and translational investigations.

## 2. Results and Discussion

### 2.1. Molecular Fractionation Reveals Compositional Complexity

**Figure 1:**
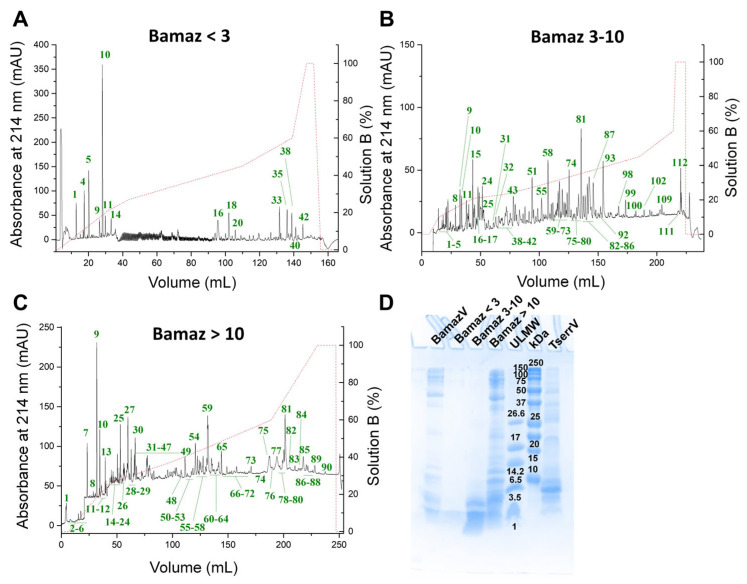
Chromatographic (**A**–**C**) and electrophoretic (**D**) profiles of *Brotheas amazonicus* venom and its components. Venom fractions enriched in <3 kDa (**A**), 3–10 kDa (**B**), or >10 kDa (**C**) components were submitted to reversed-phase chromatography on a C18 column. Samples were eluted using an acetonitrile gradient (dotted red line, displayed on the secondary *y*-axis) and monitored at 214 nm using the Äkta Pure system (Cytiva, Marlborough, MA, USA). (**D**) Tris-Tricine SDS-PAGE of *B. amazonicus* venom and its fractions. Protein gel bands were stained with 0.2% Coomassie Phastgel Blue R-350. Abbreviations: BamazV, *B. amazonicus* venom; ULMW, ultra-low molecular weight marker; TserrV, *T. serrulatus* venom.

Each fraction of BamazV (<3 kDa, 3–10 Da, and >10 kDa) was submitted to reversed-phase chromatography for the isolation of venom components and further structural characterization (Figure 1). The fractions Bamaz < 3, Bamaz 3–10, and Bamaz > 10 were resolved into at least 42, 114, and 90 subfractions, respectively. Therefore, venom fractionation following pre-ultrafiltration enhanced peptide resolution by ~3.5 times, as estimated by the increase in the number of resolved RP-HPLC subfractions relative to whole BamazV fractionation [19]. Based on protein abundance recovered from each subfraction and the predicted component size, structural characterization was conducted by N-terminal sequencing, MALDI-TOF MS, and/or MALDI-TOF MS/MS.

The fraction Bamaz 3–10 probably contains the most diverse components (Figure 1B). This diversity may originate from multiple mRNA transcripts or from a single precursor undergoing extensive post-translational processing, thereby increasing venom complexity [20,21]. Indeed, more than 300 components have been reported for *T. serrulatus* and *T. obscurus* venoms, of which approximately 80% appear to undergo some degree of proteolytic processing [4]. Based on comparative RP-HPLC profiles previously established for *T. serrulatus* venom (TserrV) [19], potassium channel toxins (KTx) are expected to elute near subfractions 31–52 in the BamazV fractionation, whereas sodium channel toxins (NaTx) are expected near subfractions 55–87 (Figure 1B).

Smaller peptides, including anionic peptides, non-disulfide bridge peptides, antimicrobial peptides, hypotensins, and peptides of still unknown function [4], may be represented by subfractions 1–14 (Figure 1A) and 1–26 (Figure 1B). In addition, the fraction Bamaz < 3 may contain highly hydrophobic peptides, eluted only at high organic solvent concentrations (subfractions 33–42, Figure 1A). These components may represent bona fide *B. amazonicus* venom peptides or degradation products derived from larger proteins. At this stage, it is not possible to unequivocally distinguish bona fide venom peptides from degradation products, a limitation that will require proteomic validation and transcriptomic correlation. High-molecular-weight venom proteins may constitute subfractions 84–114 (Figure 1B) and 72–90 (Figure 1C). Among these, there is possibly a predominance of enzymatic proteins such as serine and metalloproteases, hyaluronidase, lysozyme, phospholipases, cysteine-rich secretory proteins, chitinase, and amidation enzymes, whose presence (at the transcript or protein level) has already been identified in different *Tityus* venoms [22,23,24,25,26]. Notably, neurotoxins and other small peptides can also be detected in the fraction Bamaz > 10 due to nonspecific intermolecular interactions that hinder peptide passage through the cellulose membrane during the initial fractionation step. This phenomenon has been previously reported in scorpion venoms and reflects strong intermolecular associations rather than true molecular mass [19,27,28]. Consistently, Tris-Tricine SDS-PAGE of BamazV fractions reveals a marked tendency toward non-specific interactions among venom components (Figure 1D), probably driven by electrostatic and hydrophobic associations between highly basic neurotoxins and larger acidic or glycosylated venom proteins.

However, performing an initial separation by molecular mass before reversed-phase chromatography may enable the recovery and identification of a larger number of venom components when compared to conventional whole-venom fractionation [19,27,28]. Altogether, these data indicate the likely post-translational processing of venom proteins generating peptides, as well as peptide–protein interactions and co-elution phenomena in BamazV.

The predominance of enzymatic components and the reduced abundance of classical neurotoxins in BamazV offer a mechanistic basis for the typically mild and localized clinical manifestations of envenoming. Consistently, BamazV shows a poor representation of canonical neurotoxins and a markedly enzyme-enriched proteomic profile compared to TserrV (Figure 1D). TserrV is dominated by peptides shorter than 10 kDa, compatible with classical ion-channel neurotoxins, whereas BamazV displays a comparatively higher proportion of high molecular weight proteins (above 37 kDa), with many aligning with enzymatic classes, including hydrolases, transferases, and proteases. This compositional change suggests that BamazV may prioritize metabolic and catalytic functions rather than fast ion-channel neurotoxicity, reinforcing a mechanistic divergence from the neurotoxin-centered venom design characteristic of TserrV.

### 2.2. Proteomic Identification Unveils Novel Peptides

#### 2.2.1. Predominance of Low-Molecular-Mass Peptides

Subfractions exhibiting high purity degree and enough protein content for downstream structural analyses were characterized through mass spectrometry and/or N-terminal sequencing through Edman degradation. This proteomic workflow aimed to determine whether the molecular complexity of BamazV is primarily driven by low-molecular-weight peptides and to assess the degree of novelty among the identified components. No quantitative proteomic approaches were employed; therefore, the relative abundance of venom components should not be inferred from MALDI signal intensities, which mainly reflect ionization efficiency.

**Figure 2:**
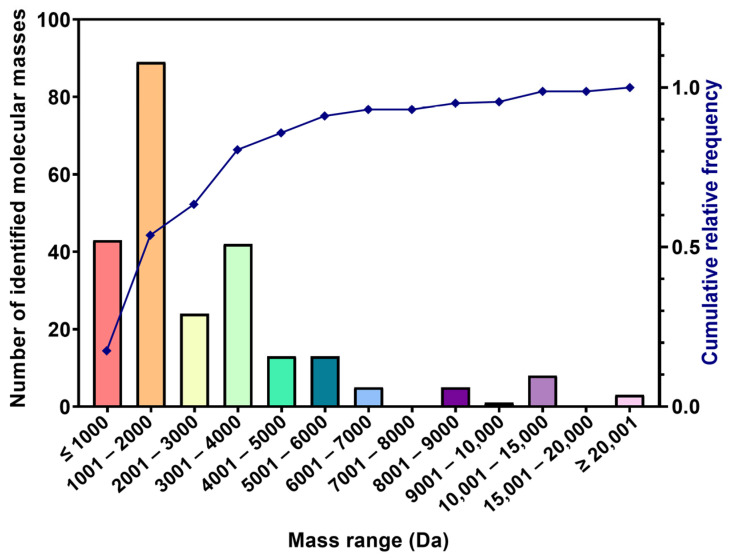
Molecular weight distribution of *B. amazonicus* venom molecular masses identified by MALDI-TOF MS. Bars represent the absolute number of detected ion signals within each mass range (Da). The overlaid line indicates the cumulative relative frequency of detected masses. Spectra were acquired in linear positive-ion mode, and peak detection reflects ionizable components above the instrument noise threshold; non-ionizable, low-abundance, or highly labile venom constituents may be underrepresented. Mass bins denote nominal *m/z* intervals and do not imply protein identity or purity.

Mass fingerprints obtained from fractions Bamaz 3–10 and Bamaz > 10 were grouped into molecular mass ranges for comparative visualization (Figure 2; Table 1).

High-molecular-mass proteins were scarcely detected, and up to 85% of the identified components presented molecular masses below 5 kDa with 53% below 2 kDa. These data demonstrate that BamazV is strongly enriched in low-molecular-mass components, a feature also observed in venoms from *Tityus* spp. [4,22,24,29,30].

Although *Brotheas* and *Tityus* belong to different families, scorpion venoms have convergently evolved for prey capture, defense and digestion [1], justifying the use of *Tityus* venoms as comparative references.

#### 2.2.2. Novelty and Database Coverage

Only 8 peptide sequences from BamazV are currently deposited in public databases, all corresponding to short fragments lacking functional annotation (Table 2).

Previously reported peptides retrieved from UniProtKB were reannotated using the standardized scorpion venom peptide nomenclature proposed by Delgado-Prudencio et al. [32], and renamed BamazP-1 to BamazP-8 to ensure nomenclature consistency and facilitate database integration. Theoretical molecular masses of these peptides were searched in the BamazV mass fingerprint. Within the limits of MALDI-TOF mass accuracy, signals compatible with BamazP-5 (1191.0 Da), BamazP-7 (1427.7 Da), and BamazP-8 (1449.7 Da) were detected in subfractions 43 (Bamaz 3–10) and 28 (Bamaz > 10) (Table 1). These sequences (except BamazP-4) share a high similarity degree and were aligned (Figure S1).

Several subfractions from Bamaz 3–10 (9 and 24) and Bamaz > 10 (subfractions 5, 9, 10, 25, 28, 29 and 30) did not show matches in public databases, probably reflecting the presence of previously unreported venom peptides. Thus, these components were subjected to automated and manual de novo sequencing (Table 3; Figure S2). While several peptides were only partially fragmented and require further validation, this may also be influenced by intrinsic molecular features, including post-translational modifications known to affect MS/MS fragmentation. Together, these findings collectively highlight the high degree of novelty and limited database coverage of BamazV peptides.

#### 2.2.3. Evidence for Proteolytic Processing and Post-Translational Modifications

Sequence similarity among BamazP-1, BamazP-2, BamazP-3, BamazP-5 and BamazP-6 suggests that these peptides may derive from a single precursor undergoing proteolytic processing, thereby increasing venom molecular diversity. Similar post-splitting mechanisms have been reported in other arthropod venoms. A well-documented example occurs in the venom gland of the ant *Neoponera villosa*, in which carboxypeptidases, aminopeptidases, and/or endopeptidases mediate post-translational processing of shared precursors (ponericins), generating multiple antimicrobial peptides with distinct biological activities [33].

The peptide BamazP-25 (VLFETKPETQG-NH_2_), identified in subfraction 30 of Bamaz > 10 (Figure S2, Table 4), was fully sequenced by combining MS/MS and Edman degradation. MS/MS data revealed a C-terminal amidation, representing the first post-translational modification (PTM) reported for a mature venom peptide from the Chactidae family, following the earlier identification of a glycosylated PLA_2_ in *Anuroctonus phaiodactylus* (Mafia scorpion, Q6PXP0) [34]. This modification is biologically relevant, as C-terminal amidation may enhance peptide stability and activity, and remains poorly documented in non-buthid scorpion venoms.

BamazP-25 shares 75% sequence similarity with the signal peptide of Ts15 rather than with its mature toxin. Ts15 is the first α-KTx from *T. serrulatus* venom that reversibly blocks rKv_1_._2_, hKv_1_._3_, Shaker IR and rKv_1_._6_ channels [35]. However, this biological activity is associated exclusively with the mature Ts15 peptide. Therefore, the observed similarity does not imply functional equivalence. Instead, it supports a hypothesis of modular evolution of venom precursors, in which signal peptide-derived sequences may be released as mature venom components following gene duplication and alternative proteolytic processing. These interpretations remain speculative and are presented as hypotheses consistent with the known structural plasticity of venom peptide precursors.

Extremely short sequences detected in Bamaz < 3 (peaks 1, 4 and 5, Table 4), hampering confident identification, whereas BamazP-9 likely corresponds to a NaTx fragment. At this stage, it is not possible to unequivocally classify these sequences as functional venom peptides or degradation fragments. Although further top-down MS/MS approaches will be required to resolve this issue, the data collectively indicate extensive proteolytic processing and pronounced molecular heterogeneity in BamazV.

#### 2.2.4. Putative Functional Classes

**Figure 3:**
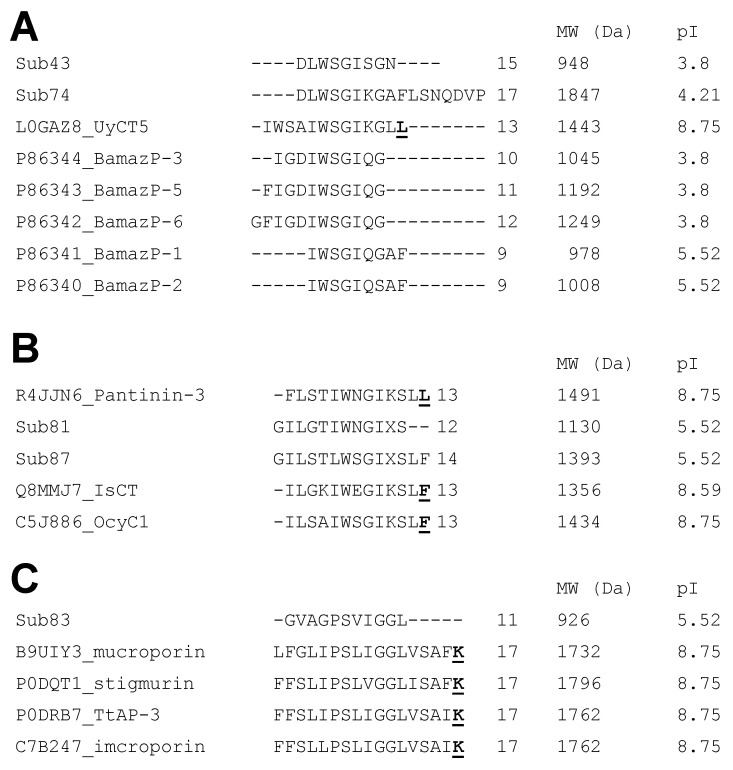
Multiple sequence alignments among components identified from Bamaz 3–10 and structurally related scorpion venom peptides available in public databases. Amidated C-termini are in bold and underlined. UniProt accession codes precede peptide names. Molecular weight (MW) and isoelectric point (pI) values were theoretically estimated using the Protparam tool (https://web.expasy.org/protparam/, accessed on 10 January 2026) without considering post-translational modifications. Sub refers to venom subfractions. (**A**) Sequence comparison among peaks 43 (BamazP-10), 74 (BamazP-15), previously reported *B. amazonicus* peptides (BamazP-1, BamazP-2, BamazP-3, BamazP-5 and BamazP-6), and the antimicrobial peptide UyCT5 from *Urodacus yaschenkoi* venom. (**B**) Comparison among the peaks 81 (BamazP-16) and 87 (BamazP-19), as well as non-buthid antimicrobial peptides pantinin-3, IsCT, and OcyC1 from *Pandinus imperator*, *Opisthacanthus madagascariensis* and *Opisthacanthus cayaporum*, respectively. (**C**) Comparison among peak 83 (BamazP-17) and the buthid antimicrobial peptides TtAP-3 (*T. trinitatis*), imcroporin (*Isometrus maculatus*), stigmurin (*T. stigmurus*) and mucroporin (*Lychas mucronatus*). Sequence similarity suggests a functional convergence towards antimicrobial structures, which justifies further experimental validation.

Based on sequence similarity, several BamazV peptides cluster with known functional classes, including antimicrobial peptides (AMPs), potassium channel toxins (KTxs), and sodium channel-active toxins. In particular, Bamaz 3–10 (peaks 81, 83, and 87) contains peptides with putative antimicrobial activity, including BamazP-16, BamazP-17, and BamazP-19, while BamazP-15 (peak 74) shows high similarity to BamazP-1 and other previously reported BamazV components (Figure 3A). BamazP-15 and BamazP-10 also share sequence similarity with the antimicrobial peptide (AMP) UyCT5 (Figure 3A) from the Indian robust scorpion *Urodacus yaschenkoi* [36], an experimentally validated antimicrobial agent active against Gram-positive and Gram-negative bacteria with low MIC (minimal inhibitory concentration) values and no detectable hemolytic activity.

Furthermore, several peptides detected in Bamaz 3–10 and Bamaz > 10 show similarity to non-disulfide bridge peptides (NDBPs) and antimicrobial peptides (AMPs) from scorpions of the Parvorder Iurida. Peaks 81 and 87 (BamazP-16 and 19) resemble pantinin-3, IsCT, and OcyC1 (Figure 3B), with all reported in Scorpionoidea venoms sharing the Iurida parvorder with *B. amazonicus* [37], and BamazP-19 was sequenced up to its putative amidated 14th residue (Figure 3B). BamazP-17 (peak 83) is similar to TtAP-3 from *T. trinitatis* (Figure 3C), while BamazP-26 (peak 13, BamazV > 10) shares similarity with the C-terminal propeptide of TsAP-1, suggesting the possible release of microbicidal fragments. Consistent with known properties of AMPs, these peptides are compatible with α-helical, cationic structures often associated with cytotoxic and immune-related functions [38,39]. Additional putative AMP-related peptide identified in BamazV > 10 is BamazP-37 (peak 77), which resembles venom protein 22.1 from *Lychas mucronatus*, a species characterized by lysine-rich precursors prone to proteolytic processing and generation of cryptic peptides [40,41].

Evidence from Edman degradation of peaks 71–72 revealed a fragment of α-amylase (BamazAmy) coeluting with BamazP-34, a component sharing sequence similarity with antimicrobial peptides. BamazP-34 displayed a well-defined N-terminal sequence of nine hydrophobic residues (AKVMLVCLA), consistent with a signal peptide-derived fragment, followed by a natural proteolytic cleavage between Ala^9^ and Ile^10^. Sequencing beyond this cleavage identified a second peptide (IXIIPGLVGGLISAXK) compatible in length and composition with linear antimicrobial peptides described in scorpion venoms. The continuity of Edman sequencing beyond this cleavage site excludes incomplete sequencing chemistry or random degradation and supports the coexistence of multiple processing products derived from a single precursor within the same chromatographic peak. The lack of classical dibasic cleavage motifs suggests non-canonical proteolytic maturation, a mechanism commonly reported for scorpion peptides [20,42]. This post-splitting diversification parallels classical examples described for scorpion venoms, including Ts19, which undergoes post-splitting to generate functionally distinct toxins [43], Ts3, which requires glycine-dependent C-terminal amidation for maturation [44], and Ts8, which is synthesized as a propeptide requiring N-terminal trimming.

In addition, a rare toxin family was detected in peak 29 (BamazP-23 and -24, Bamaz > 10), showing similarity to HtC6Tx2 of the α-C6Tx family (Table 4), presenting the pattern X_2_CX_11_CX_5_CX_11_CX_12_CX_7_CX_5_ and whose biological role remains unknown [45]. Together with the convergent biochemical evidence supporting non-canonical post-translational of an AMP precursor and the generation of a putative mature antimicrobial peptide (IXIIPGLVGGLISAXK), these findings highlight the molecular complexity of BamazV, although further analyses are required to confirm the exact proteolytic processing sites.

Finally, peak 9 (BamazP-21, Bamaz > 10) matched insectotoxin I2 from *Mesobuthus eupeus* (P15221), a sodium channel-active peptide. However, despite this identification and Edman degradation uncertainty at position 8, a Gly was inferred from mass fingerprint analysis (Table 1—1177 Da). BamazV overall lacks a pronounced neurotoxic profile when compared to buthid scorpions such as *Tityus* spp. [4], suggesting a venom strategy possibly favoring antimicrobial and modulatory peptides over potent neurotoxins.

Potassium channel toxins were detected mainly in Bamaz > 10 (peaks 28, 30, 36, and 59, Table 4), including scorpine-like peptides previously shown to dominate BamazV [19]. KTxs play a central role in prey immobilization [46] and represent valuable scaffolds for therapeutic development targeting neurological, oncological and immunological disorders [47]. The identification of two additional scorpine-like peptides, BamazScplp2 (peak 28) and BamazScplp3 (peak 30), further expands this toxin class and reinforces the pharmacological potential of BamazV components, in line with previous findings showing selective cytotoxic activity of BamazScplp1 against triple-negative breast cancer cells [18]. Notably, scorpine-like peptides exhibit multifunctionality, acting as potassium channel blockers and antimicrobial or antiparasitic agents [48,49], and the sequence assigned to BamazSclp3 may derive from proteolytic processing of a longer precursor, giving rise to a putative cryptic peptide. The presence of KTxs in high-molecular-mass fractions is probably explained by aggregation phenomena or nonspecific interactions with larger venom components, as discussed above. Sodium channel-active peptides were detected at low abundance, consistent with the absence of a pronounced neurotoxic profile.

Fragments of metalloproteinases (BamazMP-1 to 9, detected in peaks 13, 49, 66, 70, 81, and 85), phospholipase A_2_ (peak 59), chitinase (peak 89) and α-amylase (peaks 71–72) were also identified (Bamaz > 10, Table 4), indicating active proteolysis and enzymatic diversity within the venom. The presence of chitinase may be associated with catabolic processes involved in chitin degradation, potentially contributing to a digestive role of the venom, as chitinases are well-established components of digestive fluids and facilitate insect predation by degrading chitinous structures [26,50]. Chitinases were previously reported in *T. serrulatus* only at the transcriptomic level in venom glands. Together, these data support a venom architecture dominated by low-molecular-mass peptides shaped by extensive post-translational processing, with functional diversification rather than reliance on high-molecular-mass toxins.

Overall, BamazV exhibits a peptide-rich and highly novel proteomic profile, in which extensive proteolytic processing and post-translational modification contribute substantially to venom complexity and functional diversification beyond well-studied buthid species.

### 2.3. Post-Translational Diversity and Evolutionary Convergence Buthid Toxins

Post-translationally modified peptides are recurrent features in animal venoms and play fundamental roles in molecular protection, stability, folding, and enzymatic activity. In scorpion venoms, post-translational modifications (PTMs) are particularly relevant for defense-related peptides, where modifications such as C-terminal amidation increase resistance to enzymatic degradation [51] and stabilize α-helical structures, enhancing the biological activity of antimicrobial peptides [52]. As stated above, BamazP-25 represents the first evidence of C-terminal amidation in a mature chactid venom peptide. Notably, this modification also occurs in phylogenetically distant buthid and non-buthid venom peptides (Figure 3), suggesting functional convergence rather than shared ancestry. The recurrence of amidation in distant scorpion families supports the concept of independent recruitment of this PTM under similar selective pressures. Furthermore, the presence of a mature amidated scaffold in a non-buthid venom highlights its translational relevance, as peptide amidation has been repeatedly associated with improved receptor interaction, proteolytic resistance, and increased bioactivity [53,54,55]. These attributes may directly contribute to the pharmacological optimization and biotechnological usability of emerging peptides from Chactidae venom. Electrophysiological analyses revealed that Ts1-G from TserrV exhibits reduced affinity for sodium channels compared to the mature amidated Ts1, underscoring the functional importance of C-terminal amidation for toxin activity [53].

However, the novel BamazV components identified here require further investigation to clarify the prevalence, biosynthetic origin, and functional relevance of peptide amidation in Chactidae venoms. The predominance of small amidated peptides may reflect an adaptive strategy for insect predation; however, this interpretation is based on relative abundance data and functional analogy with NDBPs, and should therefore be considered as a working hypothesis in the absence of absolute quantification or direct functional validation.

Peptide C-terminal amidation in Chactidae venoms is supported by a conserved yet structurally complex biosynthetic machinery, comparable to that described for other scorpion lineages. This system operates in coordination with proprotein convertases (PC1/PC2) and carboxypeptidase E, which remove C-terminal basic residues and expose the glycine required for α-amidation. Transcriptomic analyses of the venom gland of *Anuroctonus pococki bajae* reveal the coexistence of a dual α-amidating system comprising a membrane-anchored bifunctional peptidylglycine α-amidating monooxygenase (PAM) and independently encoded, soluble monofunctional enzymes peptidyl-α-hydroxylating monooxygenase (PHMm) and peptidyl-α-hydroxyglycine lyase (PALm) [56]. These enzymes catalyze the canonical two-step reaction involving glycine α-hydroxylation followed by C–N bond cleavage, yielding the mature amidated peptide and glyoxylate. The conservation of key residues required for copper coordination and redox activity further supports the functional competence of this pathway. Phylogenetic analyses place Chactidae PALm sequences within a well-supported Iurida clade, distinct from Buthidae, indicating that diversification of amidation enzymes parallels scorpion family-level evolution while preserving catalytic function [56].

The presence of this biosynthetic machinery provides a mechanistic explanation for the occurrence of C-terminally amidated and bioactive venom peptides in Chactidae, as this post-translational modification has been experimentally shown to enhance peptide activity by increasing membrane permeabilization, accelerating bactericidal kinetics, and promoting greater conformational flexibility [57]. This interpretation is consistent with proteomic and MS/MS evidence of amidated peptides in non-buthid scorpions, including Chactidae representatives [58].

Taken together, these findings support post-translational amidation as a conserved and functionally advantageous modification in scorpion venoms, contributing to peptide diversification and ecological specialization. By extending PTM diversity to non-buthid lineages, this study reinforces the view that chactid venoms, despite their low clinical severity, have underexplored molecular characteristics with great biotechnological and pharmacological potential.

### 2.4. Enzymatic Repertoire Highlights Potential Spreading and Inflammatory Roles

The enzymatic profile of BamazV reveals a repertoire biased toward tissue diffusion and inflammatory modulation rather than extensive proteolytic or oxidative damage. Enzymatic activity assays were performed for major venom enzyme classes commonly described in animal venoms, including hyaluronidase, PLA_2_, proteases and L-amino acid oxidases (LAAOs).

#### 2.4.1. Spreading Enzymes

Hyaluronidases are key venom spreading factors that increase extracellular matrix permeability and facilitate venom biodistribution. Inhibition of *T. serrulatus* hyaluronidase has been shown to impair venom dissemination and envenoming severity [59].

**Figure 4:**
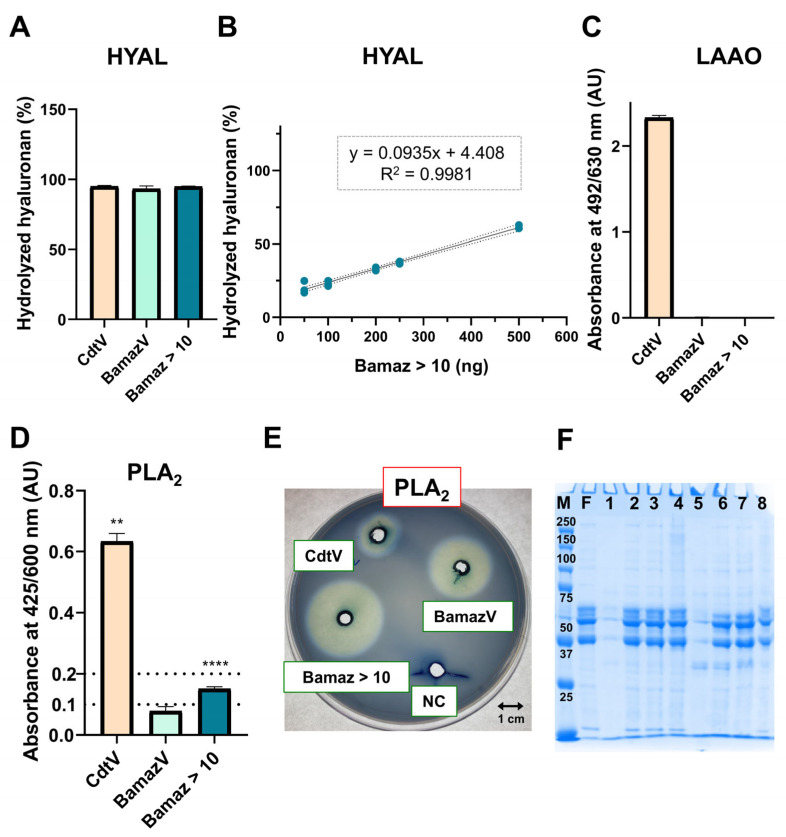
Functional enzymatic profiling of the fraction Bamaz > 10. Hyaluronidase activity was determined by turbidimetric assay to perform (**A**) a comparative screening of hyaluronidase activity among venoms and fraction, and (**B**) determination of hyaluronidase-specific activity on Bamaz > 10. (**C**) Spectrophotometric assay for determination of L-amino acid oxidase activity upon using L-leucine as substrate. (**D**) PLA_2_ activity using a chromogenic substrate (NOB). Bars represent mean ± SD (*n* = 3). (**E**) PLA_2_ activity on egg-yolk agar plate. (**F**) Fibrinogenolytic activity assay. Samples: 1, BamazV; 2, BamazV + F; 3, BamazV + F + EDTA; 4, BamazV + F + PMSF; 5, Bamaz > 10; 6, Bamaz > 10 + F; 7, Bamaz > 10 + F + EDTA; and 8, Bamaz > 10 + F + PMSF. Statistical analysis: one-way ANOVA followed by Dunnet’s test (**** *p* < 0.0001 and ** *p* < 0.01, as indicated above selected bars). Abbreviations: BamazV, *B. amazonicus* venom; CdtV, *C. durissus terrificus* venom; F, bovine fibrinogen; M, molecular weight marker; NC, negative control (buffer only).

Strong hyaluronidase activity (~94% of hyaluronan hydrolysis) was detected in both BamazV and its fraction Bamaz > 10 (Figure 4A). A protein band compatible with hyaluronidase molecular mass (~40–45 kDa) [19,60,61,62,63,64,65] was identified in Tris-Tricine SDS-PAGE (Figure 1D), and a 40,747 Da component was detected in subfraction 59 of Bamaz > 10, suggesting the presence of a hyaluronidase.

The specific activity of hyaluronidase in Bamaz > 10 (2703 TRU/mg) was approximately 4-fold higher than that previously determined for BamazV [19], indicating enzyme enrichment during our venom fractionation protocol. Although direct quantitative comparisons across venoms are limited by methodological variability, this activity exceeds values reported for several venoms of scorpions [19,22,60,61,62,63,64,65,66,67,68], spiders [69] and snakes [70].

To our knowledge, the specific activity of isolated enzymes have only been reported for *T. serrulatus* (19,900 ± 1730 TRU/mg), *Olivierus martensii* (syn. *Buthus martensii, Mesobuthus martensii*) (18,900 TRU/mg) and *Palamneus gravimanus* (6411 TRU/mg) [71,72,73].

By increasing extracellular matrix permeability, a highly active hyaluronidase may enhance the local and systemic availability of otherwise low-potency venom components, amplifying their biological effects. Such strong activity of almost 100% of hyaluronan hydrolysis, uncommon among non-buthid scorpions, suggests that diffusion efficiency may partially compensate for reduced neurotoxin potency.

#### 2.4.2. Inflammatory/Cytolytic Enzymes

PLA_2_ activity was detected in both BamazV and Bamaz > 10 using chromogenic (NOB) substrate (Figure 4D) and an egg-yolk agar plate assay (Figure 4E). Activity measured in Bamaz > 10 using the NOB substrate was approximately twice that determined for BamazV, consistent with enzymatic enrichment. PLA_2_ activity has been detected in several scorpion venoms [60,65,66,67,74,75], although enzyme isolation has only been achieved in a limited number of species, such as *Anuroctonus phaiodactylus*, *Hemiscorpius lepturus*, *Heterometrus fulvipes*, *Heterometrus laoticus*, *Pandinus imperator* and *Scorpio maurus* [76].

Until recently, *T. melici* was the only Brazilian scorpion species whose venom had been reported to exhibit detectable PLA_2_ activity [75], despite the recurrent identification of PLA_2_-like sequences in omics studies [23,24,25,75,77]. Our recent proteomic and enzymatic studies of Amazonian scorpion venoms expanded this scenario by revealing and functionally validating a Group III PLA_2_ in BamazV [19], whose partial purification is further supported here. Notably, the PLA_2_ activity observed in the present study for BamazV (10 µg), seems visually higher than that reported for *Tityus melici* venom, which required a substantially larger venom amount (165 µg) [75]. Importantly, differences in substrates and experimental conditions preclude a direct or quantitative comparison between the two venom enzymes.

Scorpion venom PLA_2_s belong to the group III-PLA_2_ family, as well as bee and lizard venom PLA_2_s [78]. They are usually composed of two polypeptides (large and small subunits derived from the same single gene) connected by a pentapeptide that is removed during protein maturation [78]. After maturation, their molecular mass ranges from 11 to 19 kDa, and their sequence generally folds into a calcium-binding loop, an antiparallel two-stranded β-sheet and three α-helices [34,66,79,80,81,82,83,84]. Here, a prominent protein band (14 kDa) can be visualized in Bamaz > 10 (Figure 1D), and MALDI-TOF MS revealed components in the 13.4–13.8 kDa range in multiple Bamaz > 10 subfractions (59, 66 and 81, Table 1), suggesting the presence of PLA_2_ in these subfractions. MALDI-TOF analysis of peak 59, the most abundant late-eluting fraction of BamazV, revealed two major components: the ∼9.2 kDa scorpine-like BamazScplp1 (UniProt C0HME9), which accounted for approximately 63% of the peak [18], and the co-purified ∼13.5 kDa phospholipase A_2_ BamazPLA_2_ (UniProt C0HMF5) [19], indicating enzymatic heterogeneity within this fraction. Minor ambiguities in Edman sequencing (peak 59, Table 4) suggest the possible coexistence of closely related PLA_2_ isoforms and may contribute to the enzymatic heterogeneity observed in this venom. Scorpion venom PLA_2_ has been associated with inflammatory, hemolytic and anticoagulant effects [66]. In BamazV, PLA_2_ activity probably contributes to local inflammation, membrane destabilization and synergism with other venom components.

#### 2.4.3. Absent or Reduced Enzymes (Oxidases/Proteases)

No detectable fibrinogenolytic activity was observed under the experimental conditions employed, even in Bamaz > 10, where high-molecular-weight proteins are enriched (Figure 4F). Although fibrinogen degradation has been previously reported for BamazV [14], its absence here and in recent studies [19] suggests intraspecific or ecological variability. Substrate specificity rather than enzyme absence also may explain this result, and future studies using broader substrate panels are warranted.

Similarly, no LAAO activity was detected in BamazV or Bamaz > 10 (Figure 4C). Moderate LAAO activity reported previously required substantially higher venom concentrations (5-fold higher) [19], suggesting that this enzyme is either present at very low levels or subject to ecological or physiological regulation.

#### 2.4.4. Ecological and Functional Interpretation

Instead of rapid systemic toxicity, BamazV appears to favor venom strategies centered on efficient diffusion, localized tissue effects and inflammatory modulation. The reduced abundance or undetectable levels of oxidases and proteases, enzymes commonly associated with extensive tissue damage and digestion in highly predatory and clinically relevant scorpions, may reflect an ecological specialization of *B. amazonicus,* potentially favoring prey immobilization [6] rather than rapid lethality. Although this interpretation is based on relative compositional and functional data and lacks absolute quantification, transcriptomic support, and direct functional assays, it is presented as a working hypothesis grounded in our experimental findings. In summary, the enzymatic repertoire of BamazV supports a distinct ecological and functional profile among non-buthid scorpions, emphasizing venom efficiency rather than clinical severity.

### 2.5. Limited Cross-Recognition of B. amazonicus Venom and Its Fractions by Commercial Scorpion Antivenom

**Figure 5:**
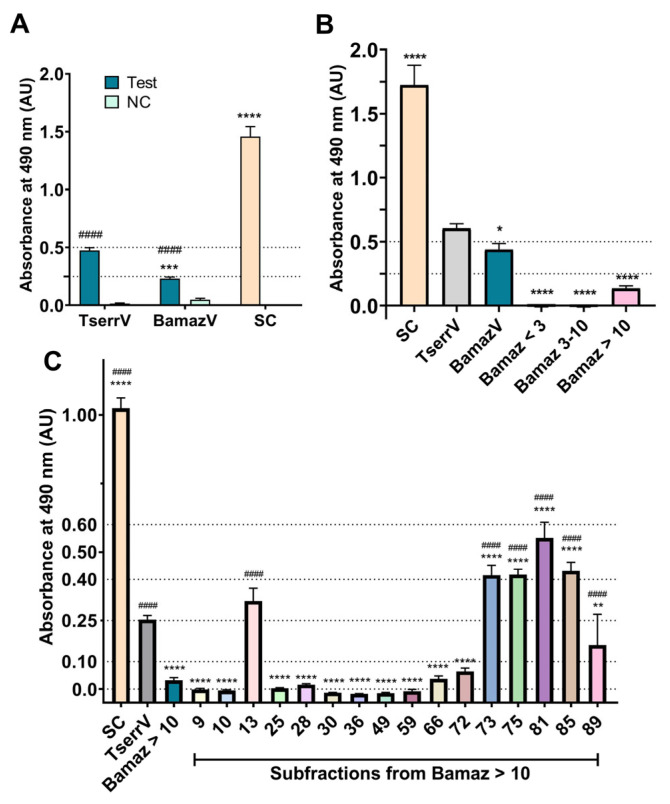
Immunorecognition of *B. amazonicus* venom (**A**) and its fractions (**B**), as well as subfractions from Bamaz > 10 (**C**) by commercial scorpion antivenom. Microplate was coated with 2 µg of venoms, fractions, or subfractions and incubated with commercial antivenom. Secondary antibody control (SC): 1 µL of non-immunized horse serum, used to confirm secondary antibody activity. Negative control (NC, panel A): commercial antivenom replaced with non-immunized horse serum to determine nonspecific binding. Absorbance was read at 490 nm. Data are shown as mean ± SD of technical triplicates. (**A**) Recognition of BamazV and TserrV relative to NC. Statistical analysis: two-way ANOVA followed by Bonferroni’s test vs. NC (^####^ *p* < 0.0001) and one-way ANOVA followed by Dunnett’s test vs. TserrV (**** *p* <0.0001 and *** *p* < 0.001). (**B**) Recognition of fractions Bamaz < 3, Bamaz 3–10, Bamaz > 10. Statistical analysis: one-way ANOVA followed by Dunnet’s test vs. TserrV (**** *p* < 0.0001, * *p* <0.05), and (**C**) recognition of subfractions from Bamaz > 10. Statistical analysis: one-way ANOVA followed by Dunnet’s test vs. TserrV (**** *p* < 0.0001 and ** *p* <0.01) and vs. Bamaz > 10 (^####^ *p* < 0.0001, as indicated above selected bars). Abbreviations: BamazV, *Brotheas amazonicus* venom; TserrV, *Tityus serrulatus* venom.

The recognition of *B. amazonicus* venom (BamazV) and its fractions by commercial scorpion antivenom was evaluated by indirect ELISA (Figure 5A). Nonspecific binding, verified using negative control serum (NC), indicated baseline recognition by antibodies from non-immunized horses. Due to the low abundance of venom in terms of protein mass produced by scorpions [85], titration and negative control assays for BamazV fractions were not performed.

Figure 5A shows that BamazV was recognized by the commercial antivenom at significantly lower levels than TserrV, exhibiting approximately twice lower absorbance. These findings highlight that current antivenoms, optimized for neurotoxin-rich *Tityus* spp, may display reduced cross-reactivity with non-buthid venoms, which are predominantly enzymatic, such as BamazV, without necessarily implying clinical severity. There are only two antivenoms produced in Brazil to treat all scorpion envenoming cases: (i) the scorpion antivenom produced using TserrV as the immunizing antigen, and (ii) the antiarachnid antivenom produced using the TserrV and the venoms from the spiders *Loxosceles gaucho* and *Phoneutria nigriventer* [86]. However, the treatment of scorpion envenoming victims in the Brazilian Amazon has been shown to be refractory to the available commercial antivenoms [6,87,88,89,90]. Nishikawa et al. [10] showed that an antivenom produced using a mixture of venoms from *T. serrulatus* and *T. bahiensis* could not recognize *B. amazonicus* venom proteins in an immunoelectrophoresis assay. Furthermore, bi or monovalent antivenoms prepared with these same *Tityus* venoms revealed low titration against *B. amazonicus* venom [10].

To date, there are no reports in the literature of the recognition of BamazV and its components by a commercial scorpion antivenom. BamazV was subdivided into three molecular weight ranges: <3 kDa, 3–10 kDa and >10 kDa. Recognition of these fractions by the commercial scorpion antivenom was also evaluated by indirect ELISA (Figure 5B). Only high-molecular-weight proteins (>10 kDa) were recognized. These components probably include large protein families, such as phospholipases A_2_, proteases, hyaluronidases and L-amino acid oxidases, which share structural similarities and immunogenic epitopes with large components from TserrV [91].

On the other hand, low molecular weight proteins (less than 10 kDa) showed minimal recognition, consistent with lower immunogenicity due to their smaller size [91], and greater difficulty in producing immunoglobulins to neutralize their effects on envenoming. Furthermore, minimal amino acid substitutions on *B. amazonicus* venom proteins compared to toxins from *T. serrulatus* venom may be enough to alter their three-dimensional conformation and hamper the recognition by the *T. serrulatus* antivenom.

The limited recognition of BamazV and its fractions suggests evolutionary divergence between Chactidae (*B. amazonicus*) and Buthidae (*Tityus*) families, which has practical implications for antivenom development. Notably, BamazV displays a LD_50_ approximately 78-fold higher than TserrV (90.909 mg/kg versus 1.160 mg/kg), and BamazV is usually considered non-toxic and non-medically important [9,10]. Envenomings by *B. amazonicus* account for only 3.3% of scorpion sting cases reported in the Manaus region (Amazonas state, Brazil), reinforcing that *Tityus* spp. are the primary cause of clinically relevant envenomings in the Brazilian Amazon [7,88,89].

Despite its low medical relevance, BamazV represents a promising source of biologically active macromolecules with potential biotechnological applications. Previously reported activities include analgesia, fibrinogen degradation, and anti-insect activity [6,14,92]. These findings emphasize that venoms with limited clinical importance may nevertheless contain compounds of interest for pharmacological and biotechnological research.

### 2.6. Antivenom Recognition Correlates with Enzyme-Rich Subfractions

Although *B. amazonicus* is not considered a medically relevant scorpion, evaluating antivenom recognition provides a valuable framework to assess the molecular biases of current antivenoms and to understand how enzymatic-dominant venoms are perceived by antibody repertoires optimized against neurotoxic buthid species.

The commercial scorpion antivenom effectiveness against Bamaz > 10 subfractions was evaluated by indirect ELISA (Figure 5C). Subfractions 13, 81, 85 and 89, which are enriched in enzymatic components (e.g., metalloproteases), were preferentially recognized. This preferential recognition is consistent with the higher immunogenicity of large, conserved venom enzymes, which present multiple accessible epitopes and therefore dominate antibody recognition. Because ELISA assays reflect antibody binding rather than functional neutralization (antivenom efficacy), the observed reactivity should be interpreted as immunogenicity, consistent with the well-documented dominance of large, conserved venom enzymes in humoral immune responses.

Peak 59 was not efficiently recognized, which may be explained by partial epitope masking due to the high proportion of the scorpine-like peptide BamazScplp1 [18,19], potentially limiting antibody accessibility to BamazPLA_2_ and the putative hyaluronidase present in this fraction. This interpretation remains associative and highlights the influence of the relative abundance of components on immunodetection.

Although peaks 73 and 75 were highly recognized, their molecular composition remains insufficiently characterized, precluding a detailed discussion. Nonetheless, their strong immunoreactivity suggests the presence of conserved antigenic determinants, possibly associated with enzymatic or structural venom proteins.

On the other hand, the low immunogenicity of short venom peptides, especially potassium channel toxins, probably accounts for the reduced antibody titers and limited neutralization capacity previously reported [10]. Due to their small size and the limited complexity of their epitopes, these peptides often induce antibody binding without effective functional neutralization, which may contribute to non-neutralizing interactions by the antivenom. It is important to emphasize that the low reactivity of the antivenom should not be equated with biological irrelevance, since short venom peptides and other low molecular weight components may exhibit limited immunogenicity while maintaining significant biological, pharmacological, and biotechnological potential.

Even within the same genus, sequence divergence and variability in antigenic epitopes among *Tityus* spp. have been shown to limit antivenom efficacy, leading to refractoriness in treatment [7]. If such antigenic divergence restricts cross-reactivity among closely related buthid species, this limitation is probably exacerbated in venoms from phylogenetically distant non-buthid scorpions, reinforcing the need for regionally and taxonomically adapted antivenoms for Amazonian scorpion envenoming. Collectively, these findings indicate that current antivenoms, optimized for neurotoxin-rich *Tityus* venoms, may display reduced effectiveness against enzyme-dominant venom profiles such as that of *B. amazonicus*. Thus, antivenom recognition in this context should not be interpreted as a measure of clinical efficacy, but rather as an indicator of the antigenic composition, molecular accessibility, and evolutionary conservation of venom components.

### 2.7. Integrative Interpretation: B. amazonicus Venom as an Enzyme-Dominant, Reduced-Neurotoxic Proteome

Taken together, these results position *B. amazonicus* as an informative representative of non-buthid scorpions with an enzyme-dominant and reduced-neurotoxic profile. Rather than prioritizing rapid lethality mediated by ion-channel toxins, the venom composition suggests an alternative functional strategy, potentially emphasizing tissue diffusion, inflammatory modulation, and prey immobilization.

The predominance of enzymatic proteins, coupled with a comparatively low abundance of classical neurotoxins, suggests a venom architecture adapted to functional efficiency rather than acute toxicity, aligning with the ecological requirements of non-buthid scorpions. In this context, the ecological niche of *B. amazonicus,* a forest species that inhabits leaf litter and fallen trunks, provides a coherent framework for interpreting the observed venom composition. A predominantly enzymatic and weakly neurotoxic venom profile is compatible with a lifestyle centered on predation of invertebrates in forest floor microhabitats, where localized tissue effects, prey immobilization, and facilitation of digestion may be more advantageous than rapid systemic lethality.

Furthermore, the identification of novel amidated low-mass peptide expands the known structural diversity of scorpion venom scaffolds, highlighting BamazV as a source of underexplored components with potential biotechnological and pharmacological relevance. In summary, this integrative analysis reinforces the ecological and functional uniqueness of BamazV within the broader context of scorpion venoms.

## 3. Materials and Methods

### 3.1. Venom and Its Fractions

Venom (BamazV) was milked from adult *B. amazonicus* specimens collected in Manaus, Amazonas, Brazil (03°04′34″ S; 59°57′30″ W), under permit No. 56748-1 of the Brazilian Biodiversity Information and Authorization System (SISBIO), and registered in the National System for Management of Genetic Heritage and Associated Traditional Knowledge (SISGEN, Ministry of the Environment) under No. A4A9FDD. Venom was extracted by low-voltage electrical stimulation of the telson, pooled, lyophilized, and stored at −20 °C until use. The pooled venom sample was obtained from 16 adult specimens (nine males and seven females). All extractions were performed on the same day, within a single collection period, by the same trained collaborator using a standardized low-stress protocol. Each specimen was subjected to three brief electrical stimulations at 9 V, the lowest effective voltage, applied exclusively to the telson to induce venom release; after the third stimulation, animals were immediately returned to their natural habitat. All selected specimens were electro-stimulated and contributed to the pooled venom used in this study.

BamazV was fractionated by ultrafiltration into >10 kDa, 3–10 kDa, and <3 kDa fractions, designated as Bamaz > 10, Bamaz 3–10, and Bamaz < 3, respectively. Crude BamazV (40 mg) was dissolved in 10 mL ultrapure water and centrifuged at 8000× *g* at 4 °C for 10 min; the supernatant was collected, and the pellet was resuspended and centrifuged under the same conditions three times. All supernatants were pooled and subjected to ultrafiltration using Amicon^®^ Ultra-15 Centrifugal Filter Ultracel^®^ 10K and 3K membranes (MilliporeSigma, Darmstadt, Germany). The resulting fractions were used for downstream analyses [18]. While some low-molecular-mass peptides were observed in the Bamaz > 10 fraction, reflecting the inherent limitations of ultrafiltration in fully separating venom components by size, this approach nonetheless provided effective enrichment of both low- and high-molecular-mass components and resulted in an increased number of resolved peaks in subsequent RP-HPLC analyses.

### 3.2. Tris-Tricine SDS-PAGE

Reduced and denatured BamazV (~40 µg of soluble protein) and its fractions (Bamaz < 3, Bamaz 3–10 and Bamaz > 10, 20 µg) were run in Tris-Tricine SDS-PAGE using 16.5% and 5% resolution and stacking gels, respectively [93]. They were compared to the electrophoretic profile of TserrV (50 µg) and to the Ultra-low Range Molecular Weight Marker (1.060–26.600 Da, M3546, Sigma, Saint Louis, MO, USA) and Precision Plus Protein Dual Color Standards (10–250 kDa, #1610374, BioRad, Hercules, CA, USA). Although protein bands were stained with 0.2% Coomassie Brilliant Blue R-350 (PhastGel^®^ Blue R-350, 17-0518-01, Pharmacia, Uppsala, Sweden), the gel image was acquired in black and white (Gel Doc™ EZ Gel Documentation system, BioRad, Hercules, CA, USA), artificially colored, and analyzed using ImageLab™ software version 5.2.1. (BioRad, Hercules, CA, USA). Protein content was relatively estimated by its absorbance at 280 nm using the NanoDrop™ 2000 Spectrophotometer (Thermo Fisher Scientific, Waltham, MA, USA). Although UV absorbance has limitations when applied to complex mixtures, it was chosen for its minimal sample requirement and compatibility with small-volume, low-yield venom fractions. A constant extinction coefficient (ε_280_ = 1.0) was used exclusively for relative quantification, not for absolute protein determination, and all readings were conducted in technical triplicate to maintain reproducibility.

### 3.3. B. amazonicus Venom Fractionation by Reversed-Phase Chromatography

Bamaz < 3, Bamaz 3–10 and Bamaz > 10 were submitted to fast liquid protein chromatography (FPLC) prior to structural analysis of their components. The reversed-phase chromatographic conditions applied to the Bamaz > 10 fraction followed a previously validated protocol established by our group [18], whereas the acetonitrile gradients used for the Bamaz < 3 and Bamaz3–10 fractions were empirically adjusted to optimize peak resolution. For this, each fraction (430–800 µg) was separated through reversed-phase chromatography on a C18 column (10.0 × 250.0 mm, 5 µm, 300 Å, Jupiter^®^ C18, Phenomenex, Torrance, CA, USA) previously equilibrated with 0.1% trifluoroacetic acid (TFA). Samples were eluted by a segmented acetonitrile gradient (0–80%) in solution B (80% acetonitrile in 0.1% TFA; 0–100% B) at a flow rate of 1 mL/min. Processes were monitored at 214 nm (Äkta pure system, Cytiva, Marlborough, MA, USA). All subfractions were lyophilized and kept at −20 °C until used in further analyses. Prior to the following steps, subfractions were dissolved in ultrapure water and protein content was relatively estimated at 280 nm using the NanoDrop™ 2000 Spectrophotometer (Thermo Fisher Scientific, Waltham, MA, USA).

### 3.4. Identification of B. amazonicus Venom Components

#### 3.4.1. Mass Spectrometry

BamazV subfractions were analyzed at the Chromatography and Spectrometry Center (CENACROESP, School of Pharmaceutical Sciences of Ribeirão Preto, University of Sao Paulo). Samples (2–20 µg) were mixed 1:1 (*v*/*v*) with either sinapinic acid (SA) for proteins > 10 kDa or α-cyano-4-hydroxycinnamic acid (CHCA) for peptides < 10 kDa, both prepared in 0.1% TFA and 50% acetonitrile, and spotted onto a polished steel MALDI (Matrix Assisted Laser Desorption/Ionization) target plate for solvent evaporation at room temperature. MALDI TOF/TOF analyses were performed using an Ultraflex II system (Bruker Daltonics GmbH, Bremen, Germany). Mass spectra were acquired in positive ion mode, using reflector mode for peptides (<3 kDa) and small proteins (3–10 kDa), and linear mode for larger proteins (>10 kDa). Spectra were accumulated over 500–2500 laser shots per spectrum at single positions, with each sample spotted in at least two technical replicates at randomized target locations to ensure reproducibility. Instrumental performance parameters are described in the Supplementary Material (File S1). Data were processed using flexAnalysis v.3.4.76.0 and DataAnalysis 4.0 (Bruker Daltonics GmbH, Bremen, Germany). Automated de novo sequencing was performed with PEAKS 12.5 (Bioinformatics Solutions Inc., Waterloo, ON, Canada), considering results with average local confidence (ALC) higher than 70%, while all fragmentation spectra were also manually inspected and validated using the MS-product tool (https://prospector.ucsf.edu/prospector/cgi-bin/msform.cgi?form=msproduct, accessed on 25 February 2024). Sequences were screened for annotation against Scorpiones (taxonomy ID: 6855) and *Tityus* (taxonomy ID: 6886) reference datasets

#### 3.4.2. N-Terminal Sequencing

Subfractions corresponding to well-resolved peaks from C18 reversed-phase chromatographies, selected based on detectable UV absorbance and sufficient yield, were submitted to N-terminal sequencing using an automated PPSQ-33A protein sequencer (Shimadzu Corporation, Kyoto, Japan) through Edman degradation method [94]. Identified sequences were analyzed by Protein BLAST (BLASTp) via the NCBI web interface against the non-redundant database, prioritizing Scorpiones (taxonomy ID: 6855) and *Tityus* (taxonomy ID: 6886) (accessed on 10 January 2026). When no significant matches were obtained, searches were expanded to the Arachnida dataset (taxonomy ID: 6854). N-terminal sequences of newly identified BamazV components are currently being deposited in the UniProt Knowledgebase [95].

### 3.5. Evaluation of the Enzymatic Activity of BamazV and Bamaz > 10

#### 3.5.1. Sample Preparation

About 1 mg of BamazV, Bamaz > 10, and *Crotalus durissus terrificus* venom (CdtV, positive control) were dissolved in ultrapure water (300 µL) and centrifuged (10,000× *g*, 4 °C, 10 min). The pellet was discarded and total soluble protein was estimated by absorbance at 280 nm using the NanoDrop™ 2000 Spectrophotometer (Thermo Fisher Scientific, Waltham, MA, USA). Enzymatic activities were calculated relative to the positive control, and the blank (buffer only) was used as the negative control condition. Due to the extremely limited amount of BamazV available, a negative control using inactivated venom or venom fraction could not be performed. All assays were carried out in triplicate. Statistical analyses were performed using GraphPad Prism 6.0.1 (GraphPad Software, San Diego, CA, USA) with one-way ANOVA followed by Dunnett’s test for multiple comparisons. Results are expressed as the mean ± standard deviation and were considered statistically significant when *p* < 0.05.

#### 3.5.2. PLA_2_ Enzymatic Activity

Firstly, PLA_2_ enzymatic activity was evaluated through the hydrolysis of egg-yolk in agar plate [96]. Plate was prepared by dissolving 0.3 g of bacteriologic agar (NCM0214A, Neogen, Lansing, MI, USA) in phosphate-buffered saline (PBS, 18.9 mL), followed by cooling to ~55 °C. Then, 50 mM CaCl_2_ (0.1 mL) and egg-yolk suspension (1 mL) were added, mixed, and allowed to solidify. Samples (20 µg of CdtV, and 10 µg of BamazV and Bamaz > 10) were applied and plate was incubated at 37 °C for 24 h. After incubation, plate was stained with 0.01% Stains-all^®^ (E9379, Sigma, Saint Louis, MO, USA) for 2 h. PLA_2_ activity was also colorimetric evaluated using the 4-nitro-3-(octanoyloxy)benzoic acid substrate (NOB, BML-ST506-0050, Enzo Life Sciences, Farmingdale, NY, USA) as [97] adapted by [98]. Samples (20 µg of CdtV, BamazV and Bamaz > 10, in triplicate) were incubated in 50 mM Tris-HCl containing 150 mM KCl and 10 mM CaCl_2_ (pH 7.5) with 500 µM NOB at 37 °C for 2 h. Absorbance was measured at 425/600 nm using a microplate reader Sunrise™ (Tecan, Männedorf, Switzerland).

#### 3.5.3. L-Amino Acid Oxidase Activity

L-amino acid oxidase activity assay was performed in triplicate as previously described for Kishimoto and Takahashi [99]. Samples (20 µg of CdtV, BamazV and Bamaz > 10), 5 mM L-leucine (L-8000, Sigma, Saint Louis, MO, USA), 2 mM o-phenylenediamine (OPD, P9029, Sigma, Saint Louis, MO, USA), and 1 U/mL horseradish peroxidase (P6782, Sigma, Saint Louis, MO, USA) were incubated in 100 mM Tris-HCl (pH 8.0) buffer at 37 °C for 1 h. Reaction was stopped with 2 M H_2_SO_4_, and absorbance was measured at 492/630 nm using the microplate reader Sunrise™ (Tecan, Männedorf, Switzerland).

#### 3.5.4. Hyaluronidase Activity

Hyaluronidase activity turbidimetric assay [100] was performed in triplicate adapted to a microplate. Samples (20 µg of BamazV, Bamaz > 10 and CdtV) were incubated with hyaluronan (10 µg, H1876, Sigma, Saint Louis, MO, USA) in 0.2 M sodium acetate buffer containing 0.15 M NaCl (pH 6.0) at 37 °C for 1 h. Following this, reaction was stopped by adding 2.5% cetyltrimethylammonium bromide (CTAB) in 2% NaOH, and samples turbidity was measured at 400 nm by the microplate reader Sunrise™ (Tecan, Männedorf, Switzerland).

The hyaluronidase specific activity of Bamaz > 10 was determined by incubating hyaluronan with different amounts of Bamaz > 10 (50–500 ng). In this case, sample turbidity was measured using the Synergy H1 Hybrid Multi-Mode microplate reader (BioTek Instruments, Winooski, VT, USA). The Turbidity Reducing Units (TRUs) are the quantity of protein necessary to hydrolyze 50% of substrate and the specific activity is TRU per mg of protein.

#### 3.5.5. Fibrinogenolytic Activity

Fibrinogenolytic activity of BamazV and Bamaz > 10 was carried out according to modifications on Edgar and Prentice method [101]. Samples (15 µg) were incubated in 100 mM Tris-HCl (pH 8.0) in the presence or absence of protease inhibitors (20 mM EDTA or PMSF) at 37 °C with continuous agitation at 300 rpm (ThermoMixer^®^ C, Eppendorf, Germany) for 30 min. Following this, fibrinogen from bovine plasma (30 µg, F8630, Sigma, Saint Louis, MO, USA) was added to each reaction mixture, and samples were incubated under the same conditions for 4 h. Reactions were stopped by adding denaturing buffer (60 mM Tris-HCl, 10% glycerol, 10% β-mercaptoethanol, 2% SDS, 0.05% bromophenol blue, pH 6.8), and heating at 100 °C for 5 min. Fibrinogen hydrolysis was evaluated by 10% SDS-PAGE [102], run at 100 V. Precision Plus Protein™ Dual Color Standard (#161-0374, Bio-Rad, Hercules, CA, USA) was used as molecular weight marker, and gel bands were stained with Coomassie Brilliant Blue R-250 (Cod. 1021, Vetec Química Fina Ltda., Duque de Caxias, Brazil). Gels were acquired in black and white using a Gel Doc system and Image Lab™ software version 5.2.1 (Bio-Rad, Hercules, CA, USA). Control reactions included fibrinogen alone, venom alone, or venom fraction alone, all submitted to the same conditions.

### 3.6. Evaluation of the Recognition of BamazV and Its Fractions and Subfractions by Commercial Scorpion Antivenom

The recognition of BamazV and its fractions and subfractions by the commercial scorpion antivenom was performed through indirect ELISA [103] and compared to TserrV. BamazV fractions were obtained according Section 3.1. The subfractions evaluated in this assay were obtained from the fractionation of Bamaz > 10 by reversed-phase chromatography (Section 3.3).

Microplate was sensitized with 2 µg of each sample or TserrV. A control using non-immunized horse serum (1 µL, H0146-5 mL, Sigma, Saint Louis, MO, USA) was included to confirm the proper functioning of the peroxidase-conjugated anti-horse secondary antibody. The experiment was performed as previously described [103] with unique modifications in the wells blocking-buffer concentration (5% MPBS, phosphate-buffered saline with milk, blotting-grade blocker—1706404, Bio-Rad, Hercules, CA, USA) and the primary antibody composition (scorpion antivenom, Instituto Butantan, batch 220170, São Paulo, Brazil).

Results were analyzed by two-way ANOVA followed by the Bonferroni’s test for multiple comparisons, using GraphPad Prism 6.0.1 (GraphPad Software, San Diego, CA, USA) when comparing venom with its negative control. Other comparisons were performed by one-way ANOVA, followed by Dunnett’s test for multiple comparisons. Results were considered statistically significant when *p* < 0.05. The negative control of the experiment was performed by substituting the commercial scorpion antivenom with non-immunized horse serum (1:100 in 1% MPBS, H0146-5 mL, Sigma, Saint Louis, MO, USA).

## 4. Conclusions

This study provides the first comprehensive characterization of the venom composition of the Amazonian non-buthid scorpion *B. amazonicus*, establishing a fundamental reference for future toxicological, ecological, and biotechnological investigations. Our integrative analytical strategy reveals a venom proteome dominated by enzymatic components and comparatively poor in classical neurotoxins, distinguishing *B. amazonicus* from the neurotoxin-rich profiles typically described for medically relevant buthid scorpions. The low medical relevance of *B. amazonicus* envenoming is consistent with a predominantly enzymatic venom architecture, supporting the notion that venom composition reflects ecological function rather than clinical severity.

The prevalence of enzymes associated with dissemination and inflammation, particularly hyaluronidase and phospholipase A_2_, corroborates a functional venom architecture oriented towards tissue diffusion, local modulation, and immobilization, rather than rapid lethality. This enzymatic predominance, along with the reduced representation of ion-channel toxins, highlights an alternative venom strategy that extends current paradigms of scorpion venom function beyond neurotoxicity-centered models and positions *B. amazonicus* as a valuable comparative model for understanding venom diversification in non-buthid lineages.

**Figure 6:**
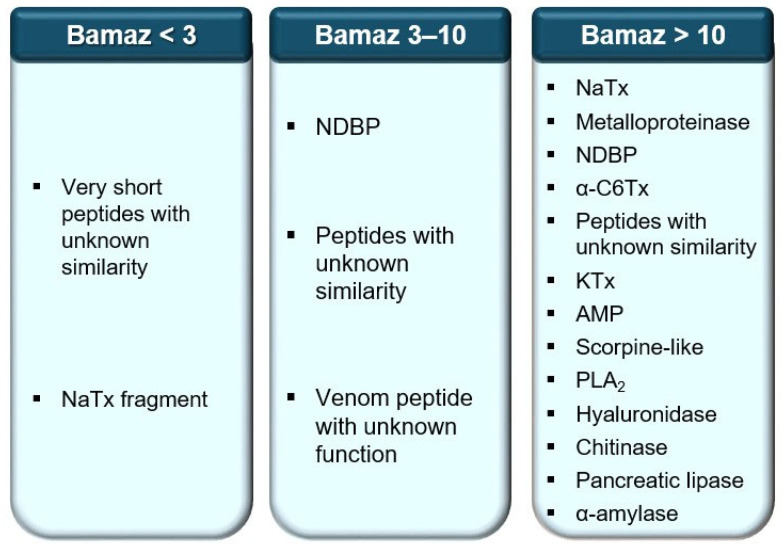
The main venom components inferred from each *B. amazonicus* venom fraction. Abbreviations: AMP, antimicrobial peptide; KTx, potassium channel toxin; NaTx, sodium channel toxin; NDBP, non-disulfide bridge peptide; PLA_2_, phospholipase A_2_.

Methodologically, the incorporation of an ultrafiltration step prior to chromatographic separation significantly improved the detection and resolution of venom components, demonstrating a robust approach to expanding venom databases of poorly studied taxa. For clarity, the main classes of venom components inferred for each molecular fraction are summarized in Figure 6. The high proportion of unannotated sequences discovered here underscores the substantial molecular diversity that remains unexplored in non-buthid scorpion venoms and reinforces their potential as reservoirs of novel bioactive structures. In this context, the sequencing of more than 40 venom peptides by MS/MS and Edman degradation establishes one of the most detailed peptidomic datasets currently available for a non-buthid scorpion species. Nevertheless, this study is limited by the lack of functional assays for several newly identified components and by the absence of in vivo validation, which should be addressed in future investigations.

Finally, the limited recognition of enzymatically predominant subfractions by commercially available antivenoms emphasizes the taxonomic and compositional constraints of current antivenom formulations, suggesting that enzyme-rich, neurotoxin-poor venoms may be insufficiently represented in existing immunization mixtures. This finding reinforces the need for venom profiles based on regional information to guide future antivenom development and optimization. Collectively, these findings position BamazV as a biologically distinct and conceptually informative system, advancing our understanding of venom evolution, functional diversification, and the translational challenges associated with antivenom design beyond buthid-centric frameworks.

## Figures and Tables

**Table 1:** MALDI-TOF mass fingerprint of peptide- and protein-enriched fractions (Bamaz 3–10 and Bamaz > 10) from *B. amazonicus* venom.

Fraction	Subfraction	*m*/*z* *
3–10 kDa	8	688.0; 726.4; 766.5; 792.4; 845.4; 865.4; 909.0; 942.6; 966.5; 1005.7; 1049.7; 1129.7; 1157.8; 1181.8; 1225.8; 1269.8; 1313.9; 1357.9; 1397.9; 1446.0; 1490.0; 1528.0; 1556.1; 1572.0; 1600.1; 1618.1; 1644.1; 1660.1; 1688.2; 1704.1; 1732.2; 1748.2; 1792.1; 1837.2
	9	866.5; 950.5; 972.5; 988.4; 1340.5; 1356.5; 1362.5; 1378.5; 1394.5; 1421.7
	24	790.5; 812.4; 828.4; 834.4; 850.4; 866.4; 877.1; 881.3; 919.5; 938.5; 957.5; 1075.7; 1110.7; 1132.7; 1148.7; 1163.7; 1189.6; 1215.9; 1230.7; 1237.8; 1253.8; 1387.8; 1403.7; 1413.7; 1423.8; 1651.9; 1673.9; 1689.8; 1705.8; 1711.8
	43	877.5; 887.7; 897.6; 913.6; 935.6; 951.6; 957.6; 973.6; 979.7; 993.8; 1002.7; 1084.7; 1126.8; 1191.0; 1213.0; 1228.9; 1247.9; 1269.8; 1275.8; 1285.8; 1402.9; 1413.9; 1420.9; 1440.9; 1442.9; 1458.9; 2013.5; 2058.6; 2072.5; 2169.7; 2994.8; 3321.4; 3321.7; 3337.4; 3337.7; 3463.8; 3464.1; 3634.3; 3634.6; 3704.7; 3720.5; 3720.8; 4159.9
	51	3003.0; 3304.4; 3359.9; 3445.8; 3671.7; 4154.7; 5567.7; 5556.8; 8357.6
	58	3072.1; 3089.1; 5727.7; 5742.9; 5754.9; 8617.5; 5731.1; 5744.0; 5757.9 (^+2^); 8617.2
	74	3302.2; 3055.5; 3968.9; 4064.0; 4673.4
	79	3413.9; 3643.6; 3655.8
	81	3089.1; 3089.3; 3378.9; 3379.2; 3394.0; 3596.6; 3610.6; 3845.4; 4179.2
	85	3421.0; 3433.0; 4319.1; 4333.1
	93	3878.8; 3894.7; 4063.1
>10 kDa	5	800.3
	9	794.5; 816.4; 832.4; 1165.7; 1177.7
	25	739.4; 761.4; 777.4; 993.6; 1304.7; 1417.7; 1455.7; 2275.4; 2407.4; 2468.8 (^+2^)
	28	1427.7; 1449.7; 2316.3; 2331.1; 2353.1
	29	993.6; 1246.6; 1355.3 (^+2^); 1415.7; 1439.7; 1455.7; 2479.6; 2494.5; 2694.7; 2709.6; 2857.7; 2872.7; 3243.7 (^+2^)
	30	1247.7; 1269.7; 1275.7; 1285.6; 1388.7; 1399.7; 1416.8; 1433.8; 1436.8 (^+2^); 1439.7; 1454.7; 1512.8; 1563.8; 2382.7; 2562.5; 2620.5; 2686.7; 2857.8; 2872.7
	59	3888.1; 4086.4
		6791.8; 9192.6; 13,382.9; 13,568.1; 13,582.6; 13,791.7
		27,155.2; 27,384.1; 40,747.1
	66	3325.3; 3335.6; 3350.6; 3367.6; 3789.8; 4270.8; 6250.0
		5565.5; 6252.9; 6597.9; 8099.3; 8108.8; 13,567.8; 13,583.8
	81	4271.1; 4287.0; 5043.5; 5059.6; 5048.8; 5064.2; 5488.5; 13,581.5
	85	no results

* All values represent *m/z*. When the ion charge state is ≥2, the corresponding charge is indicated in parentheses (^+2^) after the *m/z* value. Only reproducible peaks consistently detected across at least two acquisitions were included. Samples from Bamaz 3–10 and Bamaz > 10 fractions were diluted in sinapinic acid (SA) or α-cyano-4-hydroxycinnamic acid (CHCA) matrices and analyzed by MALDI-TOF mass spectrometry. The broad distribution of low- and high-molecular-weight signals highlights the compositional complexity of *B. amazonicus* venom and guided subsequent sequencing and immunoreactivity analyses.

**Table 2:** Previously reported *B. amazonicus* venom peptide fragments retrieved from UniProtKB.

UniProt ID ^#^	Previous Nomenclature *	Updated Nomenclature **	Sequence 1234567890123	Theoretical Molecular Mass (Da) ^##^
P86341	BaP-1	BamazP-1	IWSGIQGAF	978
P86340	BaP-2	BamazP-2	IWSGIQSAF	1008
P86344	BaP-3	BamazP-3	IGDIWSGIQG	1045
P86339	BaP-4	BamazP-4	IIDFIPQIE	1087
P86343	BaP-5 ^&^	BamazP-5	FIGDIWSGIQG	1192
P86342	BaP-6	BamazP-6	GFIGDIWSGIQG	1249
P86338	BaP-7 ^&^	BamazP-7	VAIRIIWSDIQD	1429
P86337	BaP-8 ^&^	BamazP-8	ISDDIQSIIQGIF	1449

^#^ All sequences were annotated as fragments in UniProtKB. ^##^ Molecular masses were calculated assuming unmodified N- and C-termini. ^&^ Peptide detected in this study. * First peptide designation proposed by Ireno et al. [31]. ** Updated nomenclature follows the standardized scorpion venom peptide nomenclature proposed by Delgado-Prudencio et al. [32].

**Table 3:** Putative peptide sequences from *B. amazonicus* venom inferred by automated de novo MS/MS analysis ^#^. (No significant matches were found when these sequences were searched against public protein databases).

Subfraction	Automated de Novo Sequenced Peptide ^&^	Deep Novo Score (%)	ALC (%)	Precursor		Precursor Mass Error (ppm) ^##^	PTM **
				*m/z*	*z*		
3–10 kDa							
9	Q(+42.01)HGCGRAPT(−18.01)	77	77	950.48	1	56.8	Ac, D
24	SET(−18.01)ST(−18.01)PRS	76.1	76.1	828.41	1	35.8	D
24	FEFMWT(−18.01)P(−0.98)	70.2	70.2	938.52	1	100.1	Am, D
24	AAS(−18.01)EAALLLNR	81.1	81.1	1110.69	1	61.4	D
>10 kDa *							
9	T(−17.03)PKRSLQ(−18.01)	77.5	77.5	794.46	1	6.6	Amm, D
25	E(−18.01)PRFLP(−0.98)	86	86	739.42	1	−8.1	Am
29	CSGQTQFLVYEY(−18.01)	75	75	1419.70	1	50.2	D

Abbreviations: Ac, acetylation (N-term); ALC, average local confidence; Am, amidation; Amm, ammonia-loss (typically associated with N-terminal fragmentation); D, dehydration; PTM, post-translational modification. ^#^ Only sequences with Deep Novo and ALC scores ≥ 70% were considered. ^##^ Higher mass errors reflect low signal intensity and partial fragmentation typical of complex venom mixtures. * Peptides detected in the fraction Bamaz > 10 probably reflect co-elution or association with larger venom components during ultrafiltration. ** Reported PTMs are inferred from mass shifts and may partially reflect experimental or ionization-related artifacts. ^&^ Mass shifts within sequences indicate inferred modifications.

**Table 4:** Venom components from *B. amazonicus* venom partially characterized through Edman degradation sequencing and similarity-based annotation.

Subfraction	Toxin Name	Protein Family	Sequence	Similarity [Accession Number]
<3 kDa				
1	-	-	LP !	*
4	-	-	FGDS !	*
5	-	-	F	*
10	BamazP-9	NaTx	WAAIWXAW	putative Td8 [Q1I163|*T. discrepans*]
3–10 kDa				
43	BamazP-10	-	DLWSGISGN	BaP-3 [P86344|*B. amazonicus*]; BaP-5 [P86343|*B. amazonicus*])
51	BamazP-11 BamazP-12	-	YIPQDRFINWPVRGNPGVVHLHQ ! VGDEWTGRDGD	* transketolase 1 [GFR20058.1|*Trichonephila clavata*]
58	BamazP-13	-	YIPQDDFFNNPVVGGNNPVVFHL	*
67	BamazP-14	-	YIAELNNYVXPLTGIYXILA	*
74	BamazP-15	-	DLWSGIKGAFLSNQDVP	Venom peptide 1 (BaP-1) [P86341|*B. amazonicus*]
81	BamazP-16	NDBP	GILGTIWNGIXS	Pantinin-3 [R4JJN6|*Pandinus imperator*]
83	BamazP-17	NDBP	GVAGPSVIGGL	Peptide TtAP-3 [P0DRB7|*T. trinitatis*]
85	BamazP-18	lipase	TVWCPFKLGCMGTGTGTFPGFF !	pancreatic lipase-related protein 2-like [XP_023235945.1|*C. sculpturatus*]
87	BamazP-19	NDBP	GILSTLWSGIXSLF	Amphipathic peptide OcyC1 [C5J886|*Opisthacanthus cayaporum*]
93	BamazP-20	-	VEFPLSVLXGXIXLS	*
>10 kDa				
9	BamazP-21 BamazP-35	NaTx -	DCKYYGGXLNS RDVIES	Insectotoxin I2 (Toxin BeI2) [P15221|*Mesobuthus eupeus*] *
13	BamazMP-1 BamazMP-2 BamazP-26	MP MP NDBP	QPNFLRNYDYKKYIPNNSVSYENNGTT GFTMNKYKQPFIPNNVVVYVSGGEERG ARDREIHAQIEQ	Tcis_Metallo_12 [WDU65926|*T. cisandinus*] Tcis_Metallo_11 [WDU65925|*T. cisandinus*] TsAP-1 [S6CWV8|*T. serrulatus*]
25	BamazP-32 BamazP-33	- -	GKVGEFXVFNKQTLHGAPENAEQE LTAQKVANAAGDAYAYREYENQAQ	* *
28	BamazScplp2	Sclp	HKISKMTEGFGCMANMDTRG SKMTEGFGCMANMDTRG	Scorpine [P56972|*P. imperator*]
29	BamazP-23 BamazP-24	α-C6Tx α-C6Tx	FECEEXGNFQDPDDXSXFIXCDNNXK FECEEXGHFQDPDDXSXFIXCDNNXK (equitable isoforms)	venom peptide HtC6Tx2 [AOF40177|*Hadogenes troglodytes*]
30	BamazP-25 BamazSclp3	KTx Sclp	VLFETKPETQ**G** ! (determined through MS spectrum) GKLSKMTEGFGCMANMDVMG !	putative KTx [WLF82719|*T. melici*] Scorpine [P56972|*P. imperator*]
32	BamazP-27	-	AELSWMTEGFGA	*
36	BamazP-28 BamazP-29 BamazP-30 BamazP-31	KTx KTx KTx KTx	GLTELGVQDYICNCFPAALQRPA GLTEKGVQDYICNCFPAALQRPA GLTELNVQDYICNCFPAALQYPA GLTEKNVQDYICNCFPAALQYPA	U9-buthitoxin-Hj2a [ADY39508|*Hottentotta judaicus*] U9-buthitoxin-Hj2a [ADY39508|*Hottentotta judaicus*]
49	BamazMP-5 BamazMP-6	MP MP	TMLTGITKMYNELGARILKAGAAGNI GKRGSYFGAVICSIRVLNIEKQKKG	Tcis_Meta llo_6 [WDU65920|*T. cisandinus*] disintegrin and metalloproteinase domain-containing protein 10-like [XP_023240311.1|*C. sculpturatus*]
54	BamazP-22 BamazP-36	- -	AMVSQIPKLYKEITNMILQAVKAVGKMDMALSMGMISDFR TLXDEXDGVPRIVGRRMHEXAXKEAIDPADXTKEXGYALV	* *
59	BamazScplp1 BamazPLA_2_	Sclp PLA_2_	GLIKEQYFHKANDSLSYLIPKPVVNKLVGNAAXQMIHXIGXVQ TVWGTXWCGAGNESTDYXELGYFNDADRCCRXH	C0HME9 C0HMF5
59E	BamazP-39	-	XEICLQYFTGE	Venom protein 214 [P0CJ10|*Lychas mucronatus*]
66	BamazMP-8 BamazMP-9	MP MP	GFDXXSNIGSALREFIMSMGVATLAGQAL DLCXXTDASLMENXSYVAYAKGNYPNEVA	A disintegrin and metalloproteinase with thrombospondin motifs 5 [GBM47826.1|*A. ventricosus*]
70	BamazP-38 BamazMP-7	- MP	GILRIIWSDIRDVFGCQGLRN FWGRIAWEATEERPRCE	BaP-7 [P86338|*B. amazonicus*] astacin-like metalloprotease toxin 5 [XP_023232131.1|*C. sculpturatus*]
71–72	BamazP-34 BamazAmy	AMP Amylase	AKVMLVCLAIXIIPGLVGGLISAXK !** WVVRVYW !	Con22 precursor [L0GBQ6|*Urodacus yaschenkoi*]; ToAP3 [P0DQT2|*T. obscurus*] α-amylase-like [XP_067140601.1|*C. vittatus*]
73	-	-	#	*
74	-	-	#	*
75	-	-	#	*
77	BamazP-37	NDBP	PKKYKYK	Venom protein 22.1 [P0CJ04|*Lychas mucronatus*]
81	BamazMP-4	MP	KLIRDENQAREFHLNLDEKMVKA	disintegrin and metalloproteinase [AMO02516|*T. serrulatus*]
83	-	-	#	*
85	BamazMP-3	MP	QDVDSCNSYTRFV	astacin-like metalloprotease toxin 1 [XP_023230424|*Centruroides sculpturatus*]
89	BamazChi	Chitinase	DDVDP	probable chitinase 10 [XP_023236728|*C. sculpturatus*]

* no significant similarity found; # no sequencing signal; ! means sequence end; X is a non-determined residue; X is any amino acid except Cys; ** Underlined residues indicate the N-terminal signal peptide-derived fragment identified by Edman degradation, which showed a higher signal intensity than the continuing sequence. Modified amino acids are in bold. Multiple sequences per subfraction reflect co-elution and the limited resolving power of N-terminal sequencing for complex venom mixtures.

## Data Availability

The original contributions presented in this study are included in the article/Supplementary Material. Further inquiries can be directed to the corresponding authors.

## Supplementary Materials

The following supporting information can be downloaded at https://www.mdpi.com/article/10.3390/ijms27031475/s1.

## Abbreviations

The following abbreviations are used in this manuscript:

Ac	N-terminal acetylation
ALC	Average local confidence
Am	Amidation
Amm	Ammonia loss
AMP	Antimicrobial peptide
BamazV	*Brotheas amazonicus* venom
Bamaz < 3	*Brotheas amazonicus* venom fraction with components shorter than 3 kDa
Bamaz 3–10	*Brotheas amazonicus* venom fraction with components between 3 and 10 kDa
Bamaz > 10	*Brotheas amazonicus* venom fraction with components higher than 10 kDa
CdtV	*Crotalus durissus terrificus* venom
CTAB	Cetyltrimethylammonium bromide
D	Dehydration
EDTA	Ethylenediaminetetraacetic acid
ELISA	Enzyme-linked immunosorbent assay
F	Bovine fibrinogen
HCCA	α-cyano-4-hydroxycinnamic acid
KTx	potassium channel toxins
LAAO	L-amino acid oxidase
M	Molecular weight marker
MALDI	Matrix Assisted Laser Desorption/Ionization
MIC	Minimal inhibitory concentration
MPBS	Phosphate-buffered saline with milk
MW	Molecular weight
NaTx	Sodium channel toxins
NC	Negative control
NCBI	National Center for Biotechnology Information
NDBP	Non-disulfide bridge peptide
NOB	4-nitro-3-(octanoyloxy)benzoic acid
OPD	o-phenylenediamine
PBS	Phosphate-buffered saline
pI	Isoeletric point
PLA_2_	Phospholipase A_2_
PMSF	Phenylmethylsulfonyl fluoride
PTM	Post-translational modification
SA	Sinapinic acid
SC	Secondary antibody control
SDS-PAGE	Sodium Dodecyl Sulfate-Polyacrylamide Gel Electrophoresis
SISBIO	Brazilian Biodiversity Information and Authorization System
SISGEN	National System for Management of Genetic Heritage and Associated Traditional Knowledge
TFA	Trifluoroacetic acid
TOF	Time-of-flight
TRU	Turbidity reducing units
TserrV	*Tityus serrulatus* venom
ULMW	Ultra-low molecular weight marker
UV	Ultraviolet
