# Supplementary material for: A Hydrolase-Rich Venom Beyond Neurotoxins: Integrative Functional Proteomic and Immunoreactivity Analyses Reveal Novel Peptides in the Amazonian Scorpion Brotheas amazonicus
Source: Int J Mol Sci. 2026 Feb 2;27(3):1475. doi: 10.3390/ijms27031475 (PMC12897845; doi:10.3390/ijms27031475)
Supplement: Supplementary file 1 [file ijms-27-01475-s001.zip › Supplementary File S1.pdf]

```
BamazP-7    VAIRIIWSDIQD--  12
BamazP-8    -ISDDIQSIIQGIF  13
BamazP-6    GFIGDIWSGIQG--  12
BamazP-5    -FIGDIWSGIQG--  11
BamazP-3    --IGDIWSGIQG--  10
BamazP-1    -----IWSGIQGAF  9
BamazP-2    -----IWSGIQSAF  9
```

Figure S1. Multiple sequence alignment of previously reported *B. amazonicus* venom peptides. Peptide names follow the updated nomenclature introduced in Table 2. Amino acids with low consensus across the alignment are highlighted in red. Dashes indicate alignment gaps.

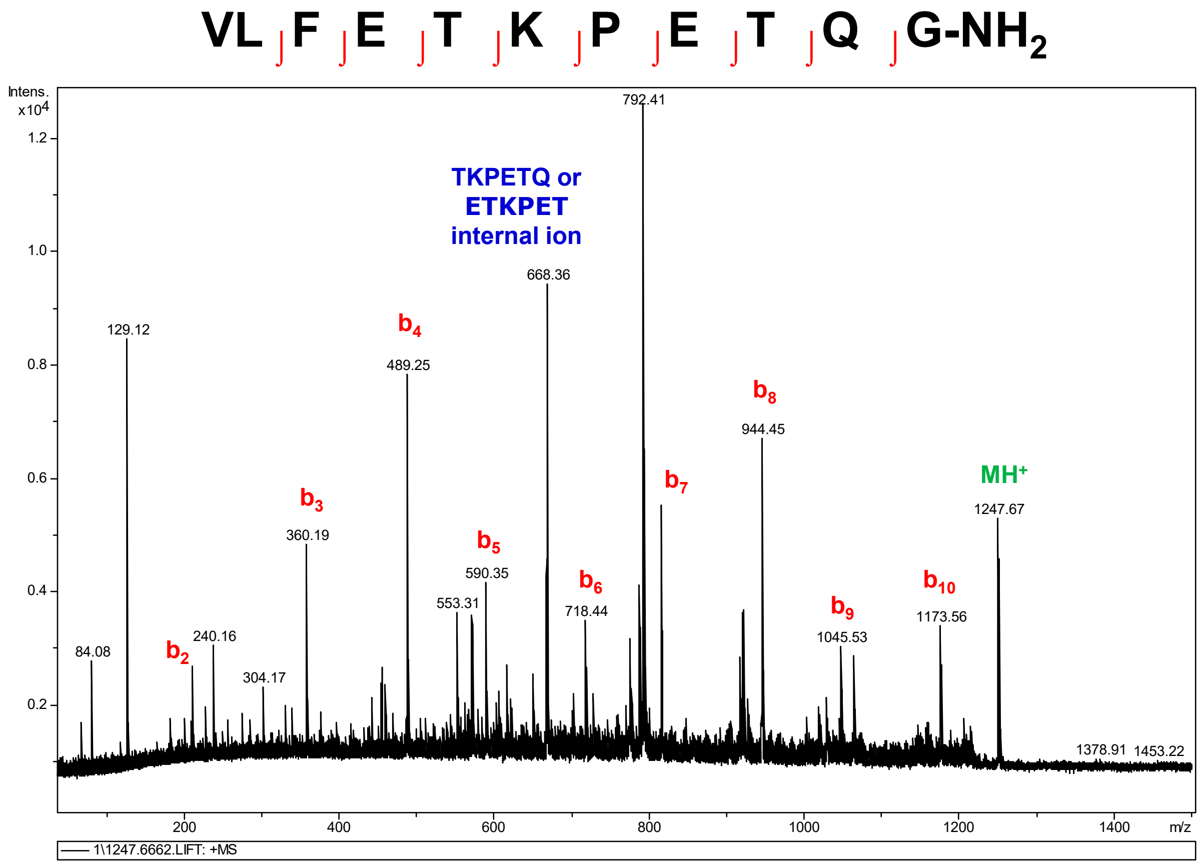

Figure S2. MS/MS spectrum of the peptide VLFETKPETQG-NH$_2$ detected in subfraction 30 from Bamaz>10. The annotated b- and y-ion series confirm sequence assignment. Theoretical monoisotopic mass: 1247.6630 Da; experimental mass (precursor m/z): 1247.6662 Da, corresponding to a 2.57 ppm mass error.

Supplementary Methods: Detailed MALDI-TOF/TOF instrumental parameters, including mass resolution, mass accuracy, and external calibration standards. MALDI-TOF/TOF analyses were performed on an Ultraflex II mass spectrometer (Bruker Daltonics GmbH, Bremen, Germany). Under the experimental conditions employed, mass resolution exceeded 14,000 for peptides (<3 kDa) and 10,000 for larger proteins (>10 kDa), with mass accuracy better than 50 ppm. External calibration was carried out using Bruker Peptide Calibration Standard II (1,000-3,500 Da), Protein Calibration Standard I (4,000-20,000 Da), and Protein Standard II (20,000-70,000 Da).
