# Supplementary figures and images for: A Hydrolase-Rich Venom Beyond Neurotoxins: Integrative Functional Proteomic and Immunoreactivity Analyses Reveal Novel Peptides in the Amazonian Scorpion Brotheas amazonicus
Source: Int J Mol Sci. 2026 Feb 2;27(3):1475. doi: 10.3390/ijms27031475 (PMC12897845; doi:10.3390/ijms27031475)

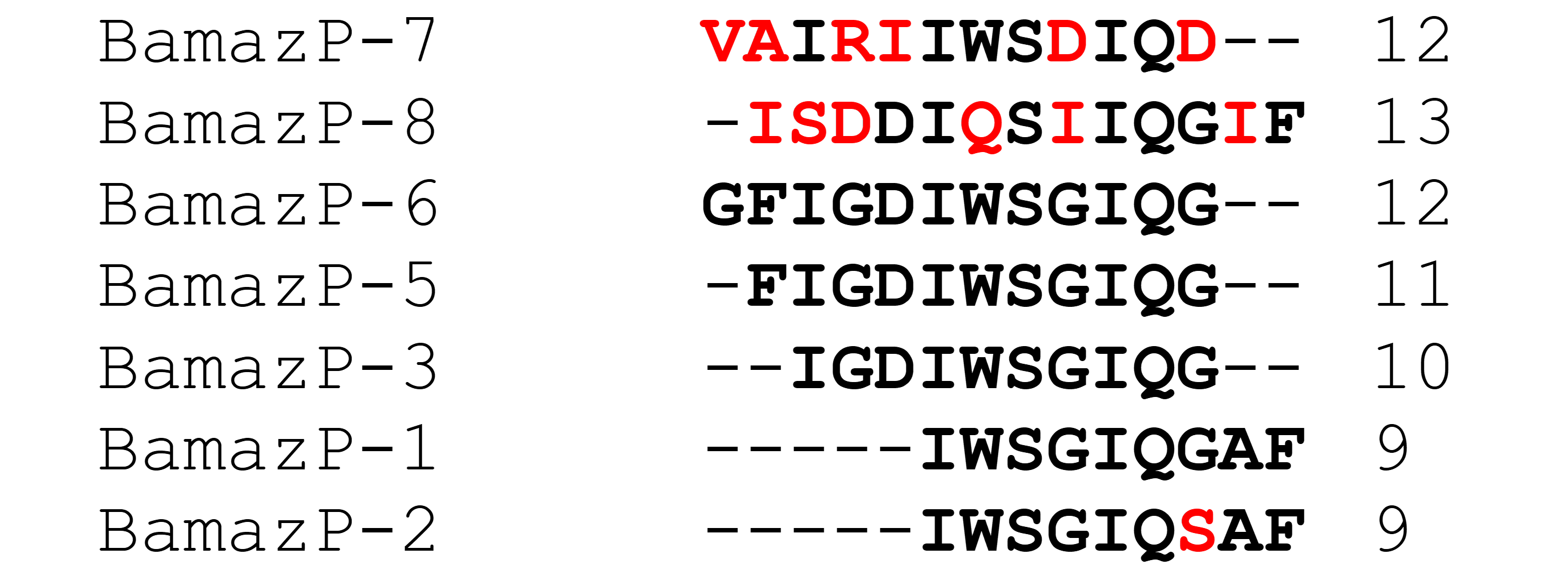

Supplement: Supplementary file 1 [file ijms-27-01475-s001.zip › Figure S1.tif]

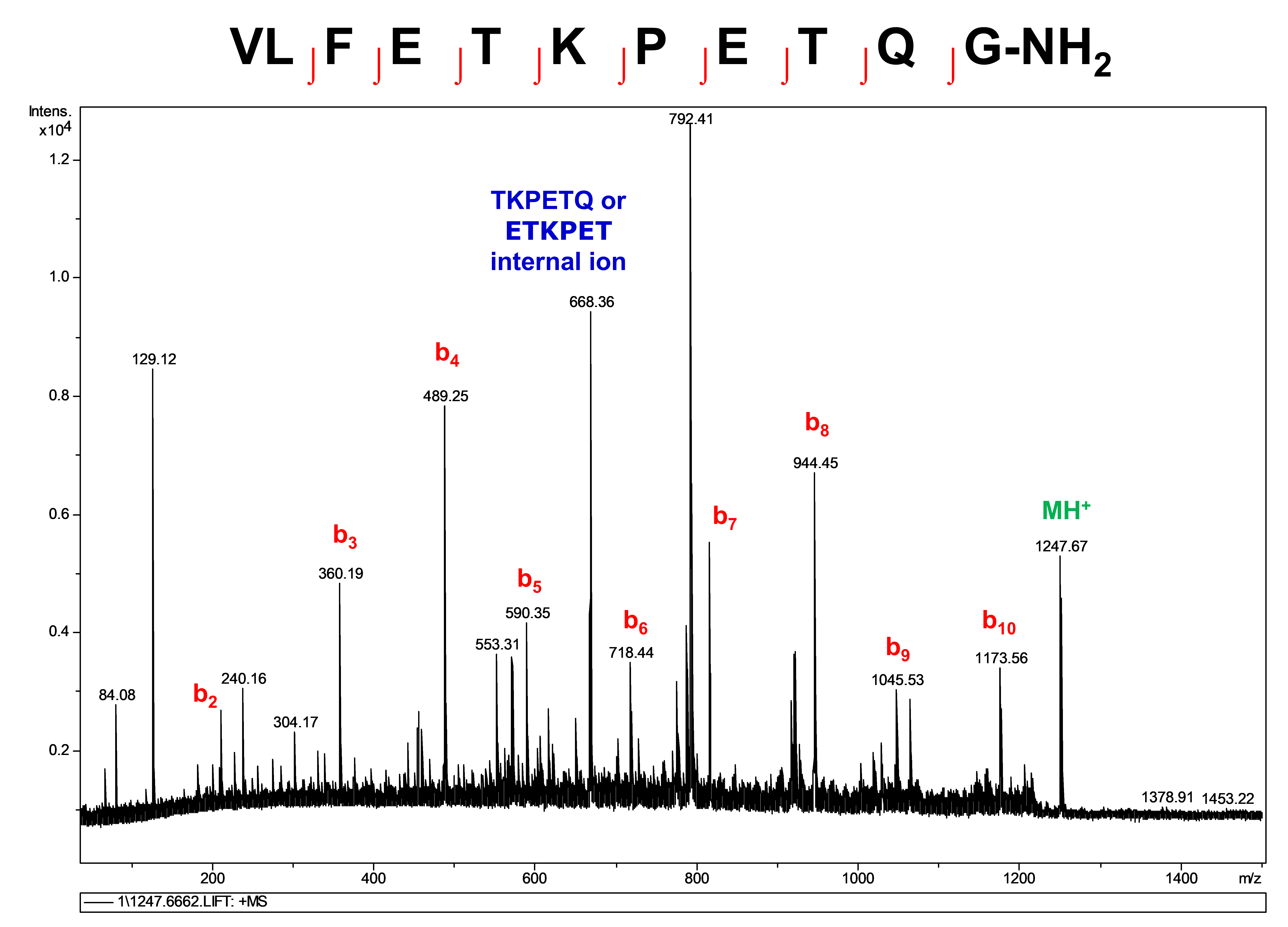

Supplement: Supplementary file 1 [file ijms-27-01475-s001.zip › Figure S2.tif]
